# Clinical Pathological Analysis of Surgically Resected Superficial Esophageal Carcinoma to Determine Criteria for Deciding on Treatment Strategy

**DOI:** 10.1155/DTE.3.211

**Published:** 1997

**Authors:** H. Makuuchi, H. Shimada, K. Mizutani, O. Chino, T. Nishi, H. Tanaka, T. Machimura, T. Mitomi, Y. Osamura

**Affiliations:** 1 Department of Surgery Tokai University School of Medicine Boseidai, Kanagawa Isehara 259-11 Japan; 2 Department of Pathology Tokai University School of Medicine Japan

## Abstract

We performed a clinical pathological study of conventionally resected superficial esophageal carcinomas since this type of lesion has been increasing, in order to develop criteria of determination for therapeutic strategies. Pathological studies were performed on specimens obtained by radical surgical resection in 133 cases of superficial esophageal cancer. Evaluation was performed in terms of the gross classification of the lesion type, depth of invasion, lymph node metastasis, vascular invasion, size of the lesion, outcome, etc. In 0-I, 0-IIc+0-IIa, and 0-III type submucosal cancer lesions the rate of metastasis to lymph nodes was more than 40%, but in 0-IIa and 0-IIb mucosal cancer cases no lymph node metastasis was observed. 0-IIc type lesions showed a wide range of invasiveness, ranging from m1 to sm3. In cases with m1 or m2 invasion, no lymph node or lymph-vessel invasion was recognized, but in m3, sm1, sm2, and sm3 cases lymph node metastasis was recognized in 12.5%, 22.2%, 44.0% and 47.4%, respectively. In 47% of lesions with a greatest dimension of less than 30 mm invasion was limited to the mucosa. Seventy-two percent of m1 and m2 cases were 30 mm in size or less. Lymph node metastasis was recognized in only 16.7% of cases less than 30 mm in size, but in cases of lesions 30 mm or more the rate of lymph node metastasis was 35.8%. 0-IIb and 0-IIa type lesions are indications for endoscopic esophageal mucosal resection (EEMR), while 0-I, 0-IIc+0-IIa, and 0-III lesions should be candidates for radical surgical resection. In the 0-IIc category, lesions in which the depression is relatively flat and with a finely granular surface are indications for EEMR, but those cases in which the surface of depression shows granules  of varying sizes should be treated with radical surgical resection. Cases of 0-IIa type 30 mm or larger in greatest dimension which have a gently sloping protruding margin shoulder or reddening should be treated with caution, but EEMR can be performed first and subsequent therapeutic strategy decided on, based on the pathological findings of the specimen.


*Diagnostic and Therapeutic Endoscopy*, 1997, Vol. 3, pp. 211–220
Reprints available directly from the publisher
Photocopying permitted by license only

© 1997 OPA (Overseas Publishers Association)
Amsterdam B.V. Published in The Netherlands
by Harwood Academic Publishers
Printed in Singapore

### Clinical Pathological Analysis of Surgically Resected Superficial Esophageal Carcinoma to Determine Criteria for Deciding on Treatment Strategy

H. MAKUUCHI[1*], H. SHIMADA, K. MIZUTANI, O. CHINO, T. NISHI, H. TANAKA, T. MACHIMURA, T. MITOMI, and Y. OSAMURA[2]

[1]*Department of Surgery, Tokai University, School of Medicine*
[2]*Department of Pathology, Tokai University, School of Medicine*

*(Received 2 November 1996; in final form 16 December 1996)*

**We performed a clinical pathological study of conventionally resected superficial esophageal carcinomas since this type of lesion has been increasing, in order to develop criteria of determination for therapeutic strategies. Pathological studies were performed on specimens obtained by radical surgical resection in 133 cases of superficial esophageal cancer. Evaluation was performed in terms of the gross classification of the lesion type, depth of invasion, lymph node metastasis, vascular invasion, size of the lesion, outcome, etc. In 0-I, 0-IIc+0-IIa, and 0-III type submucosal cancer lesions the rate of metastasis to lymph nodes was more than 40%, but in 0-IIa and 0-IIb mucosal cancer cases no lymph node metastasis was observed. 0-IIc type lesions showed a wide range of invasiveness, ranging from m1 to sm3. In cases with m1 or m2 invasion, no lymph node or lymph-vessel invasion was recognized, but in m3, sm1, sm2, and sm3 cases lymph node metastasis was recognized in 12.5%, 22.2%, 44.0% and 47.4%, respectively. In 47% of lesions with a greatest dimension of less than 30 mm invasion was limited to the mucosa. Seventy-two percent of m1 and m2 cases were 30 mm in size or less. Lymph node metastasis was recognized in only 16.7% of cases less than 30 mm in size, but in cases of lesions 30 mm or more the rate of lymph node metastasis was 35.8%. 0-IIb and 0-IIa type lesions are indications for endoscopic esophageal mucosal resection (EEMR), while 0-I, 0-IIc+0-IIa, and 0-III lesions should be candidates for radical surgical resection. In the 0-IIc category, lesions in which the depression is relatively flat and with a finely granular surface are indications for EEMR, but those cases in which the surface of depression shows granules**

*Correspondence and Reprint Address: H, Makuuchi, M.D., Department of Surgery, Tokai University, School of Medicine, Boseidai, Isehara, Kanagawa 259-11, Japan Tel: 81-463-93-1121, Fax: 81-463-95-6491

**of varying sizes should be treated with radical surgical resection. Cases of 0-IIa type 30 mm or larger in greatest dimension which have a gently sloping protruding margin shoulder or reddening should be treated with caution, but EEMR can be performed first and subsequent therapeutic strategy decided on, based on the pathological findings of the specimen.**

*Keywords:* Early esophageal cancer, endoscopic mucosal resection of esophageal cancer, superficial esophageal cancer, surgical resection of esophageal cancer, treatment strategy of esophageal cancer

### INTRODUCTION

Increasing numbers of superficial esophageal cancer lesions are being detected due to the applications of dye staining methodologies [1,2] and other improvements in endoscopic diagnosis. The total number of esophageal cancer cases treated at our institution through December 1995, totaled 957, of which 28% were superficial esophageal cancers limited to within the mucosal or submucosal layers. The increase in the detected numbers of superficial esophageal cancer lesions became most notable from around 1986, which coincided with the increasing use of direct view panendoscopy. Initially, submucosal cancers predominated amongst this group, but the increasing use of the iodine dye staining technique from around 1990 resulted in an increase in the number of mucosal cancers. With the rapid increase in the number of cases, various types of new therapeutic methods such as endoscopic esophageal mucosal resection (EEMR) [3,4] appeared, and it is now becoming increasingly important to develop criteria for the indications of such therapeutic strategies based on thorough clinical pathological analysis of superficial esophageal cancer.

We analyzed the clinical pathological feature and outcome of cases of superficial esophageal cancer that had been treated by conventional surgical resection and lymph node dissection at our institution. Based on these findings, we attempted to develop criteria for the selection of therapeutic strategy for superficial esophageal cancer.

### MATERIALS AND METHODS

### Patients

A total of 957 cases with esophageal cancer were treated at our institution from 1974 through December 1995. In 268 cases pathological evaluation showed superficial cancer with invasion extending at most to the submucosal layer. The study focused on the 133 cases of this group, excluding 126 patients treated with EEMR, 2 cases treated with transhiatal blunt resection of the esophagus without lymph node dissection, 2 cases of basaloid carcinoma, 2 cases of small cell carcinoma, 1 of carcinosarcoma, 1 of adenocarcinoma and 1 of malignant melanoma. The 133 cases treated by radical surgical resection of the esophagus with lymph node dissection consisted of 114 men and 19 women, with ages ranging from 41 to 82 (average 61.3, Table I).

### Clinical Pathological Features

In cases of multiple cancer, the most prominent lesion was considered to be the primary lesion. Clinical pathological studies were performed to determine the gross classification, size of the lesion, depth of the lesion, lymph node metastasis, vascular invasion, multiple primary lesions in the same esophagus, multiple primary cancers of other organs, and outcome based on the Guidelines for Clinical and Pathologic Studies on Carcinoma of the Esophagus of the Japan Society of Esophageal Diseases [5].

TABLE I Baseline characteristics of the 133 cases study patients

| CHARACTERISTIC | | NUMBER |
|---|---|---|
| Age at surgery (years) | | |
| | range | 41 – 82 |
| | mean | 61.3 |
| Sex | Male | 114 |
| | Female | 19 |
| Size (longest dimension of lesion) (mm) | | |
| | range | 3 – 130 |
| | mean | 33.7 |
| Gross classification | | |
| | 0-I | 16 |
| | 0-I + 0-IIc | 21 |
| | 0-IIa | 6 |
| | 0-IIa+ 0-IIc | 12 |
| | 0-IIb | 17 |
| | 0-IIc | 40 |
| | 0-IIc + 0-IIa | 5 |
| | 0-III | 16 |
| Depth of invasion | | |
| | m1 | 28 |
| | m2 | 8 |
| | m3 | 16 |
| | sm1 | 18 |
| | sm2 | 25 |
| | sm3 | 38 |
| Lymph node metastasis | | |
| | positive | 35 |
| | negative | 98 |
| Lymphatic invasion (1y) | | |
| | positive | 65 |
| | negative | 68 |
| Blood vessel invasion (v) | | |
| | positive | 34 |
| | negative | 99 |
| Multiple primary (multicentric) lesion in same esophagus | | |
| | present | 32 |
| | absent | 101 |
| Multiple primary cancer in other organs | | |
| | Double cancer | 37 |
| | Triple cancer | 9 |

### Features of Superficial Esophageal Cancer

The gross appearance of superficial esophageal cancers as observed on resected specimens was classified (Table I). When a lesion consisted of a combination of two types, the predominant type was recorded first (e.g., 0-IIc + 0-IIa). The 0-IIb category, which is unclear on conventional observation was removed from the combined classification parameters, but when a 0-IIa lesion is seen at the center of the 0-IIc lesion, it was recorded as 0-IIa + 0-IIc. When protrusion around the margin of 0-IIc lesion is present, this was recorded as 0-IIc + 0-IIa. Slightly irregular features within the 0-IIc lesion that appeared irrelevant were not included in the combined lesion classification system. The size of the lesion was expressed as the longest dimension of the lesion (in millimeters). To accurately express the depth of invasion both the mucosal layer and submucosal layer were divided into three layers. Invasion to the intraepithelial layer of the mucosal layer was expressed as ml, that to the proper mucosal layer is m2, and that to the lamina muscularis mucosae as m3, while the submucosal layer similarly divided into the sml, sm2 and sm3.

### Depth of Invasion according to Lesion Type

Depth of invasion was examined in terms of gross classification of the type of the lesion. This was performed in an attempt to show the invasive depths associated with individual types of lesion and to determine whether the depth of invasion could be estimated from the lesion type.

### Rates of Lymph Node Metastasis and Vascular Invasion according to Lesion Type

In a similar manner, the rate of lymph node metastasis, lymphatic invasion, and blood vessel invasion were evaluated in terms of the gross classification of lesion type in order to clarify the rates of metastasis and vascularization of the different lesion types.

### Rates of Lymph Node Metastasis and Vascular Invasion according to Depth of Invasion

The features of lymph node metastasis and vascular invasion were examined in terms of depth of invasion to determine whether these factors could be estimated based on the evaluation of the depth of invasion.

TABLE II Gross classification of lesion type and depth of invasion

| gross classification | m1 | m2 | m3 | sm1 | sm2 | sm3 | total |
|---|---|---|---|---|---|---|---|
| 0-I | 0 | 0 | 1 | 2 | 7 | 6 | 16 |
| 0-I + 0-IIc | 0 | 0 | 0 | 1 | 7 | 13 | 21 |
| 0-IIa | 3 | 1 | 2 | 0 | 0 | 0 | 6 |
| 0-IIa + 0-IIc | 1 | 0 | 8 | 1 | 2 | 0 | 12 |
| 0-IIb | 15 | 2 | 0 | 0 | 0 | 0 | 17 |
| 0-IIc | 9 | 5 | 5 | 11 | 6 | 4 | 40 |
| 0-IIc+0-IIa | 0 | 0 | 0 | 2 | 1 | 2 | 5 |
| 0-III | 0 | 0 | 0 | 1 | 2 | 13 | 16 |
| total | 28 | 8 | 16 | 18 | 25 | 38 | 133 |

m1: epithelial layer, m2: proper mucosal layer, m3: lamina muscularis mucosae, sm1: upper 1/3 of submucosal layer, sm2: middle 1/3 of submucosal layer, sm3: lower 1/3 of submucosal layer

### Size of Lesion According to Depth of Invasion

To determine the relationship between depth of invasion and lesion size, the longest dimension of the lesions were classified into: under 10 mm, 10 mm ≦ < 30 mm, 30 mm ≦ < 50 mm, 50 mm or more. Lesions with a longest dimension of under 10 mm are referred to as minute carcinoma. Lesions with a greatest dimension of 50 mm or more are referred to as extensive spreading type superficial cancer. Lesions up to approximately 30 mm in longest dimension can be resected in one bite with EEMR technique. The above factors are reasons for the establishment of the four groups in the study.

### Rates of Lymph Node Metastasis and Vascular Invasion and Lesion Size

The rates of lymph node metastasis and vascular invasion were evaluated in relation to the size of the lesion using the above-mentioned four groups.

### Outcome of Superficial Esophageal Cancer

The outcome of superficial esophageal cancer cases was evaluated in terms of depth of invasion and survival, excluding and also including cases of death due to other diseases, malignant or benign. The 5-year-survival was evaluated by the method of Kaplan-Meier. The 133 cases in this study were followed up thoroughly. Two other cases were excluded because they were lost to follow-up.

### Statistical Analysis

Student's t-test was used to compare the means of all variables, and the chi-square test was used to compare the prevalence of characteristics. The log-rank test was used to determine whether survival rates, as calculated by the Kaplan-Meier method, differed in any two groups. All analyses were based on the intention-to-treat principle, and all p values were two sided. The level of significance was $p < 0.05$.

### RESULTS

### Classification of Type of Lesions and Depth of Invasion

All except one of the 0-I and 0-I+0-IIc cases were submucosal cancer (Table II). The single exception was a case of m3 invasion. All 0-III type lesions were submucosal cancers and about 2/3 of the 0-II type lesions were mucosal cancers. All 0-IIa and all 0-IIb lesions were mucosal cancers, as were approximately

TABLE III Gross classification of lesion type in relation to lymph node metastasis and vascular invasion

| gross classification | number of lesions | lymphnode metastasis (%) | lymphatic invasion (%) | blood vessel invasion |
|---|---|---|---|---|
| 0-I | 16 | 8 (50.0) | 14 (87.5) | 5 |
| 0-I+0-IIc | 21 | 9 (42.9) | 16 (76.2) | 11 |
| 0-IIa | 6 | 0 | 1 (16.7) | 0 |
| 0-IIa+0-IIc | 12 | 2 (16.7) | 4 (33.3) | 2 |
| 0-IIb | 17 | 0 | 0 | 0 |
| 0-IIc | 40 | 5 (12.5) | 13 (32.5) | 4 |
| 0-IIc+0-IIa | 5 | 4 (80.0) | 5 (100.0) | 2 |
| 0-III | 16 | 7 (43.8) | 12 (75.0) | 10 |
| total | 133 | 35 (26.3) | 65 (48.9) | 34 |

TABLE IV The depth of invasion in relation to lymph node metastasis and vascular invasion

| depth of invasion | number of lesions | lymphnode metastasis (%) | lymphatic invasion (%) | blood vessel invasion (%) |
|---|---|---|---|---|
| ml | 28 | 0 | 0 | 0 |
| m2 | 8 | 0 | 0 | 0 |
| m3 | 16 | 2 (12.5) | 5 (31.3) | 3 (18.8) |
| sml | 18 | 4 (22.2) | 13 (72.2) | 2 (11.1) |
| sm2 | 25 | 11 (44.0) | 19 (76.0) | 9 (36.0) |
| sm3 | 38 | 18 (47.4) | 28 (73.7) | 20 (52.6) |
| total | 133 | 35 (26.3) | 65 (48.9) | 34 (25.6) |

a half of the 0-IIc lesions. In mixed type lesions, 3/4 of 0-IIa+0-IIc were mucosal cancer, but 100% of 0-IIc+0-IIa were submucosal cancers. In comparison to the distribution for all superficial cancers there was a significantly greater number of submucosal cancers ($p < 0.01$) in 0-I, 0-I+0-IIc and 0-IIc+0-IIa cases, whereas in 0-IIa and 0-IIb cases there was a significantly greater number ($p < 0.01$) of mucosal cancers. All ml cases were subtypes of the 0-II type, and of these 0-IIb accounted for 53.6%. Almost all (98.1%) of mucosal cancers belonged to the 0-II category.

### Lymph Node Metastasis and Vascular Invasion according to Lesion Type

Metastasis was observed in almost half of the 0-I and 0-I+0-IIc type lesions (Table III). In these categories, as well as in the 0-III type category, there were high levels of lymph node metastasis, lymphatic invasion and blood vessel invasion. Rates were much lower in the 0-II type. In particular, in the 0-IIa and 0-IIb categories there was only a single case of lymphatic invasion (0-IIa, 16.7%) and there was no case of lymph node metastasis or blood vessel invasion. Rates for categories 0-IIc (40 cases) were also relatively low.

0-IIc + 0-IIa combined type showed much higher rates of lymph node metastasis and lymphatic invasion and blood vessel invasion than 0-IIa + 0-IIc type. Compared to the overall figures for superficial esophageal cancer, the rate of lymph node metastasis for categories 0-I, 0-I+0-IIc, 0-III and 0-IIc+0-IIa was significantly greater ($p = 0.01$) whereas categories 0-IIa and 0-IIb were significantly ($p < 0.01$) lower.

TABLE V Depth of invasion and size of lesion

| depth of invasion | range | (mean) | longest dimension of the lesion (mm) <10 | 10≦<30 | 30≦<50 | 50≦ |
|---|---|---|---|---|---|---|
| ml | 3 - 50 | (19.1) | 9 | 13 | 4 | 2 |
| m2 | 4 - 130 | (35.6) | 2 | 2 | 2 | 2 |
| m3 | 10 - 130 | (38.0) | 0 | 5 | 8 | 3 |
| sm1 | 8 - 100 | (36.1) | 2 | 7 | 3 | 6 |
| sm2 | 7 - 100 | (37.5) | 2 | 11 | 3 | 9 |
| sm3 | 12 - 96 | (38.5) | 0 | 13 | 15 | 10 |
| total | 3 - 130 | (33.7) | 15 | 51 | 35 | 32 |

TABLE VI Size of lesion in relation to lymph node metastasis and vascular invasion

| size of lesion (mm) | number of lesions | lymphnode metastasis (%) | lymphatic invasion (ly) (%) | blood vessel invasion (v) | ly or v positive |
|---|---|---|---|---|---|
| < 10 | 15 | 2 (13.3) | 3 (20.0) | 0 | 3 |
| 10≦<30 | 51 | 9 (17.6) | 23 (45.1) | 9 | 25 |
| 30≦<50 | 35 | 12 (34.3) | 17 (48.6) | 13 | 20 |
| 50≦ | 32 | 12 (37.5) | 22 (68.8) | 12 | 22 |
| total | 133 | 35 | 65 | 34 | 70 |

### Depth of Invasion in relation to Lymph Node Metastasis and Vascular Invasion

Among the ml and m2 lesions there was not a single case exhibiting lymph node metastasis, lymphatic invasion or blood vessel invasion (Table IV). Figures for all 3 parameters increased roughly in accordance with the increasing depth of invasion. The rate of lymph node metastasis for ml, m2 and m3 cases were significantly ($p < 0.01$) less than that for the overall figure for superficial esophageal cancer, whereas those for sm2 and sm 3 were significantly ($p < 0.05$) greater.

### Depth of Invasion and Size of Lesion

The size of the lesion was 3 to 50 mm in ml cases with an average of 19.1 while m2 and m3 ranged from 4 to 130 mm in longest dimension with an average of 35.6 to 38.0 mm (Table V). Most of the 15 lesions under 10 mm in size were mucosal cancer (73.3%). Among those under 50 mm there were roughly equal percentages (average 40%) of mucosal cancer. About a half of the lesions under 30 mm in size were mucosal cancer, as opposed to less than a quarter of the lesions with a size of 50 mm or more. Approximately 40% of lesions less than 30 mm in size were ml or m2 lesions.

### Lesion Size in relation to Lymph Node Metastasis and Vascular Invasion

Even when the size of the maximum dimension of the lesion was under 10 mm, lymph node metastasis or lymphatic invasion was observed in a small number of cases (13.3%, 20.0%, respectively) (Table VI). Lymph node metastasis, lymphatic invasion, and blood vessel invasion was seen in 16.7%, 39.4% and 13.6%, respectively in cases less than 30 mm in size. These three parameters tended to increase in accordance with the size of the lesion.

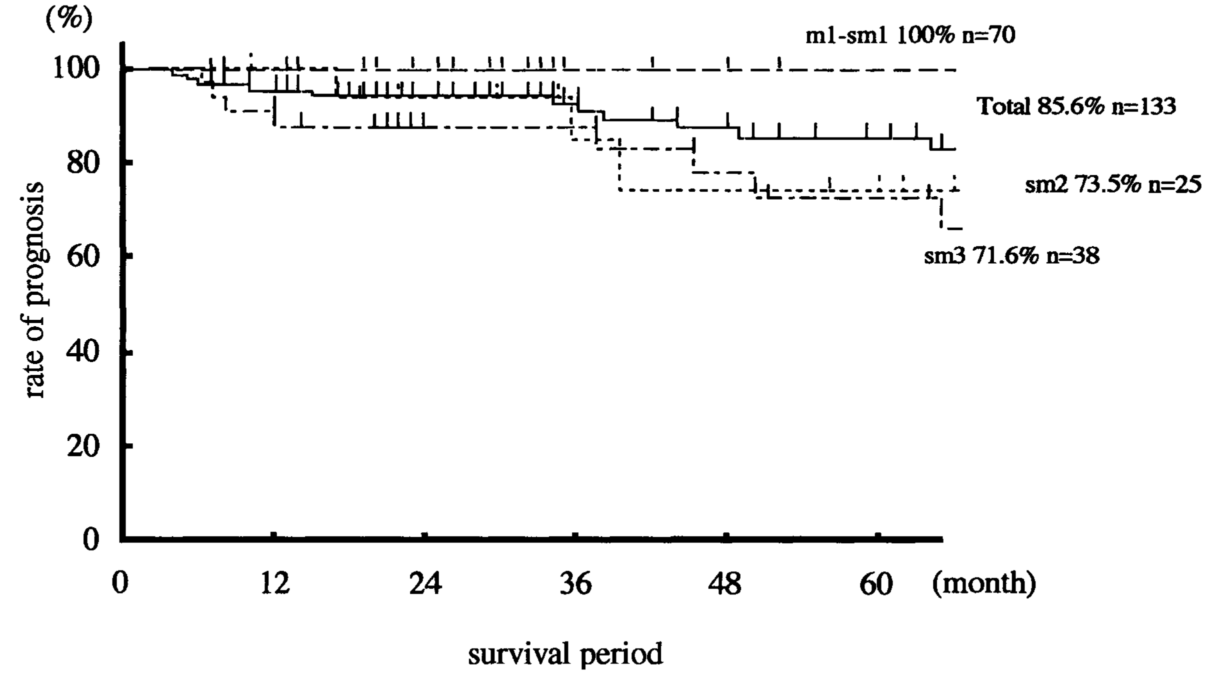

FIGURE 1 Outcome of surgically treated cases for superficial esophageal cancer (excluding cases dying from other benign or malignant disease) (Kaplan-Meier method).

### Outcome of Superficial Esophageal Cancer

As is shown in Figure 1, the overall 5-year survival for superficial esophageal cancer, excluding cases dying of other diseases or cancer of other organs, was 85.6%. Among cases with invasion extending from ml through sml no fatality due to esophageal cancer was observed, yielding a 5-year survival of 100%, which was significantly better than the 73.5% and 71.6% of sm2 and sm3 cases ($p < 0.05$) (Fig. 1). As shown in Fig. 2, the overall 5-year survival of all superficial esophageal cancer cases due to deaths from all causes was 64.6% with the figures for ml to sml being 78.4%, sm2 43.4%, and sm3 54.8%.

### DISCUSSION

The gross classification of superficial esophageal cancer specimens is based on the degree of protrusion or depression of the specimen. Those with distinct protrusions or depressions are thought to reflect a relatively advanced condition with deeper invasion and more frequent lymph node metastasis, whereas relatively flat lesions are thought to be early stage. Furthermore, comparing protruding and depressed lesions, protruding lesions which develop towards the center of the lumen are believed to have relatively shallower invasion than the depressed lesions.

The 0-I, 0-I+0-IIc and 0-III types all have distinct protrusion or depression and almost all of these categories show submucosal layer invasion. Lymph node metastasis is seen in more than 40% of such cases, while vascular invasion is seen in more than 75%, therefore in these cases radical surgical resection is indicated.

Almost all 0-IIb and 0-IIa cases were mucosal cancers, and 91.3% of them were either ml or m2. In none of these cases was lymph node metastasis

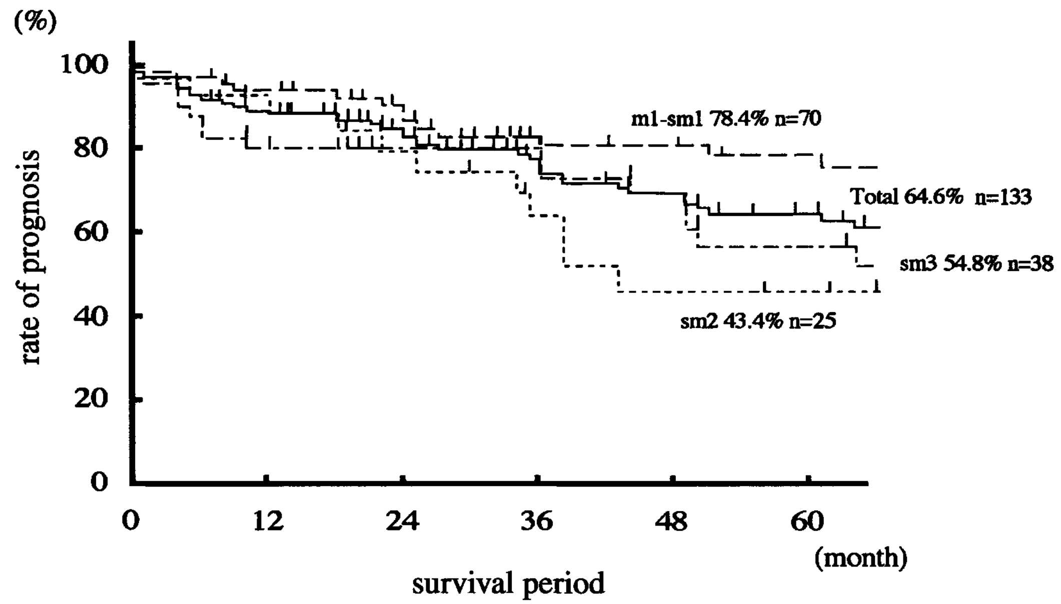

FIGURE 2 Outcome of surgically treated cases for superficial esophageal cancer (including cases dying from other benign or malignant disease) (Kaplan-Meier method).

recognized and there was only one case of lymphatic invasion. These cases are, therefore, thought to be good indications for EEMR [4]. The two m3 cases seen in type 0-IIa showed a gentle slope or reddening with a relatively large lesion. In the case of the latter, lymphatic invasion was present. It is therefore thought that cases exhibiting the latter two types of morphologies or appearance should not be included in the indications for EEMR.

The most common type lesion was 0-IIc and these seem to consist of a wide range of lesions, from early lesions with m1 invasion to more advanced sm3 invasion. Of the 40 cases in the 0-IIc category, all 5 with lymph node metastases (12.5%) had submucosal invasion. Looking at this figure from another perspective, in terms of the number of 0-IIc submucosal cancers, 5 of the 21 cases (23.8%) were found to have lymph node metastasis. Cases of type 0-IIc lesion which have m1 invasion show extremely shallow depressions which need to be distinguished from 0-IIb type lesions, while m2 lesions in this category show shallow depression with smooth fine granular surface. These cases are indications for EEMR but cases in which the granularity of the depression becomes more rough and in which the size of the granules becomes more irregular have increased likelihood for submucosal cancer, and they are therefore candidates for radical surgical resection [6]. Especially in cases in which it is difficult to decide on whether it is mucosal cancer or submucosal cancer, if the size is 30 mm or more, the likelihood of submucosal invasion is greater.

In 0-IIa+0-IIc type lesions, the 0-IIc type lesion portion seem to indicate more advanced growth. In 66.7% of cases the extent of invasion was m3. Since the rate of lymph node metastasis was fairly low (16.7%), when the largest dimension of the lesion was less than 30 mm if the 0-IIa portion was whitish with limited degree of irregularity, EEMR is considered to be indicated. The two 0-IIa+0-IIc type cases with sm2 invasion were found to have lymph node metastasis. Lesions in which the

TABLE VII Treatment strategy for superficial cancer

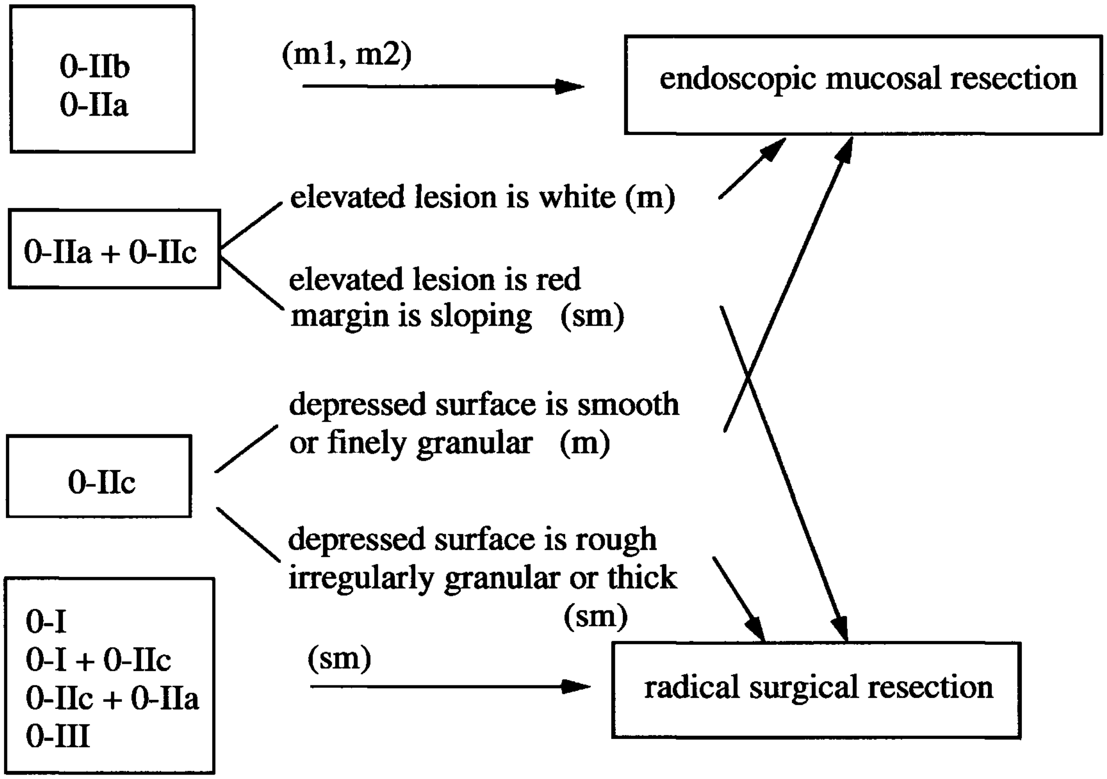

0-IIa portion had a gentle shoulder or had redness with a relatively large protrusion and lesions 30 mm or more in size in which the 0-IIc portion had irregularly sized granules were found to have submucosal invasion. In such cases radical surgical resection is indicated.

The 0-IIc+0-IIa type is considered to be a more advanced form of the 0-IIc type and the surface of the depression is thicker than in the 0-IIc type. In cases accompanied by protrusion around the entire circumference of the lesion, the depth of invasion extended to the submucosal layer in all cases, with lymph node metastasis seen in 80% and lymphatic invasion in 100%, suggesting a very poor prognosis. Even if such lesions are very small, radical surgical resection is indicated rather than EEMR. The treatment strategy for esophageal superficial cancer is shown in Table VII.

Evaluation of the depth of invasion with superficial esophageal carcinoma is being performed on the basis of the endoscopic findings and endoscopic ultrasonography [8,9]. The accuracy of such procedures is only 80%, but in cases in which the preoperative evaluation of the depth of invasion is m2, m3 or sm1, since the prognosis of sm1 cases is extremely good, it is probably better to perform EEMR and then further treatment based on the pathological results of the specimen obtained on that procedure.

Japan is now entering the age referred to as the graying of the society and superficial esophageal cancer is becoming detected with increasing frequency in elderly cases with high operative risk. We would like to suggest that EEMR should be performed whenever indicated in order to avoid the extremely high level of invasiveness of radical surgical resection.

### *Acknowledgement*

The authors are indebted to Professor J. Patrick Barron of the International Medical Communications Center of Tokyo Medical College for his review of the manuscript.

The authors also would like to thank Miss M. Iwasawa (Department of Surgery, Tokai University School of Medicine) for her help with the manuscript and statistical analysis.

### *References*

[1] Makuuchi, H. Methodology of endoscopic staining method, iodine method. In Endo, M, Ide H., ed. Endoscopic staining in early diagnosis of esophageal cancer. Tokyo: *Japan Scientific Societies Press.* 1991: 5–10.

[2] Makuuchi, H., Mitomi, T., Tajima, T., *et al.* Endoscopic diagnosis of esophageal mucosal cancer. *Stomach and Intestine* 1994; **29**: 319–326 (in Japanese).

[3] Makuuchi, H., Machimura, T. Mizutani, K., *et al.* Endoscopic mucosal resection for early carcinomas of esophagus. In Takahashi T, ed. Recent advances in management of digestive cancer. Tokyo: *Springer-Verlag*, 1993: 94–97.

[4] Makuuchi, H. Endoscopic mucosal resection for early esophageal cancer – indications and techniques. *Diag. Endosc.* 1996; **8**: 175–179.

[5] Japanese Society for Esophageal Diseases. Guidelines for the clinical and pathologic studies on carcinoma of the esophagus. *Jpn. J. Surg.* 1976; **6**: 69–78 (in Japanese).

[6] Kato, H., Tachimori, Y., Watanabe, H. *et al.* Superficial esophageal carcinoma: surgical treatment and the results. *Cancer* 1990; **66**: 2319–2323.

[7] Makuuchi, H., Machimura, T., Mizutani, K., *et al.* Treatment of mucosal and submucosal cancer in esophagus – the turning point to decide whether surgical operation or endoscopic surgery. *Jpn. J. Surg. Gastroenterol.* 1993; **26**: 2517–2521 (in Japanese)

[8] Murata, Y., Muroi, M., Yoshida, M. *et al.* Endoscopic ultrasonography in the diagnosis of esophageal carcinoma. *Surg Endosc* 1987; **1**: 11–16.

[9] Sugimachi, K. Ohno, S., Fujishima, H. *et al.* Endoscopic ultrasonography detection of carcinomatous invasion and of lymph nodes in the thoracic esophagus. *Surgery* 1990; **107**: 366–371.

